# Identification of Central Nervous System Oncologic Disease Biomarkers in EVs from Cerebrospinal Fluid (CSF) of Pediatric Patients: A Pilot Neuro-Proteomic Study

**DOI:** 10.3390/biom13121730

**Published:** 2023-11-30

**Authors:** Xhuliana Kajana, Sonia Spinelli, Andrea Garbarino, Ganna Balagura, Martina Bartolucci, Andrea Petretto, Marco Pavanello, Giovanni Candiano, Isabella Panfoli, Maurizio Bruschi

**Affiliations:** 1 Laboratory of Molecular Nephrology, IRCCS Istituto Giannina Gaslini, 16147 Genoa, Italysoniaspinelli@gaslini.org (S.S.);; 2 Department of Neurosciences, Rehabilitation, Ophthalmology, Genetics, University of Genoa, 16132 Genoa, Italy; 3 Proteomics and Clinical Metabolomics Unit at the Core Facilities, IRCCS Istituto Giannina Gaslini, 16147 Genoa, Italy; martinabartolucci@gaslini.org (M.B.);; 4 Department of Pharmacy (DIFAR), School of Medical and Pharmaceutical Sciences, University of Genoa, 16132 Genoa, Italy; 5 Department of Experimental Medicine (DIMES), University of Genoa, 16132 Genoa, Italy

**Keywords:** cerebrospinal fluid, extraventricular drainage, extracellular vesicles, exosome, microvesicle, proteomics, syntaxin-binding protein 1 (STXBP1)

## Abstract

Cerebrospinal fluid (CSF) is a biochemical–clinical window into the brain. Unfortunately, its wide dynamic range, low protein concentration, and small sample quantity significantly limit the possibility of using it routinely. Extraventricular drainage (EVD) of CSF allows us to solve quantitative problems and to study the biological role of extracellular vesicles (EVs). In this study, we implemented bioinformatic analysis of our previous data of EVD of CSF and its EVs obtained from congenital hydrocephalus with the aim of identifying a comprehensive list of potential tumor and non-tumor biomarkers of central nervous system diseases. Among all proteins identified, those enriched in EVs are associated with synapses, synaptosomes, and nervous system diseases including gliomas, embryonal tumors, and epilepsy. Among these EV-enriched proteins, given the broad consensus present in the recent scientific literature, we validated syntaxin-binding protein 1 (STXBP1) as a marker of malignancy in EVD of CSF and its EVs from patients with pilocytic astrocytoma and medulloblastoma. Our results show that STXBP1 is negatively enriched in EVs compared to non-tumor diseases and its downregulation correlates with adverse outcomes. Further experiments are needed to validate this and other EV markers in the blood of pediatric patients for translational medicine applications.

## 1. Introduction

The main causes of neurological disease in children, besides inborn or developmental brain disorders, include trauma, infection, toxic exposures, metabolic causes, inflammation [1], and tumors. As the child’s central nervous system (CNS) is a developing organ, CNS childhood diseases can lead to long-term morbidity and complex phenotypes. Given the dynamic clinical dimension of pediatric CNS diseases, the relative rarity of the single disorders, and the ethical implications of pediatric research, the availability of biomarkers is crucial for timely disease diagnosis, prognosis, stratification, and treatment response monitoring. However, the discovery of reliable protein biomarkers for pediatric CNS conditions is still in its infancy. The clinical application of cerebrospinal fluid (CSF) as a liquid biopsy has been proposed to advance the management of pediatric neurological disorders [2]. CSF analysis offers a unique window for studying pathologies and disorders of the CNS. Being in contact with the brain parenchyma is an excellent source of biomarkers of the CNS [3,4,5]. CSF is a metabolically active medium that contains proteins and other substances reflecting the cell composition of the CNS and the processes occurring inside it [6]. CSF proteome has been shown to reflect that of brain tissue [7]. CSF is mostly formed in the choroid plexus and partly in the ependyma of the ventricular system [8]. According to the recent glymphatic system concept, CSF flows [9] directionally through the CNS, clearing the interstitium from harmful proteins such as amyloid-β with a circadian rhythm [10]. Many unique proteins have been found in CSF [5,11,12,13,14]. An MS characterization of healthy normal CSF from lumbar puncture (LP) has identified 2630 proteins [12]. The CSF composition, especially its protein profile, may reflect the pathological state. The proteomic analysis of CSF has recently evidenced significant differences between the composition of CSF of patients with various CNS pathologies and healthy controls, indicating that CSF could represent an effective source of biomarkers for neurodegenerative, oncological, neurodevelopmental, and epileptic disorders [3,4,15,16]. Nonetheless, the translation from basic research to clinical applications of the identified childhood CNS disease biomarkers remains elusive.

CSF contains water, electrolytes, glucose, a few white cells, and proteins. CSF proteins can be found in the soluble fraction, or they can be kept inside the lipid bilayer of extracellular vesicles (EVs). EVs are nanovesicles released by every cell type and can be found in all bodily fluids, including CSF. EVs are heterogeneous in composition and biogenesis, often overlapping in size and biomarker content, and are secreted into the extracellular space. The latest MISEV guidelines classify EVs into exosomes (Ex, 20–150 nm, deriving from the fusion of multivesicular bodies with the cell membrane), microvesicles (Mv, 100–1000 nm, originating from the budding form the plasma membrane), and apoptotic bodies (1000–5000 nm, formed during the apoptotic program of cells). EVs carry various types of RNA, lipids, and proteins, shuttling them to recipient cells, as a means of cell-to-cell communication. EVs are being studied as potential biomarker sources for brain tumors, but also for non-oncologic brain diseases such as epilepsy [17]. Notably, similarly to what occurs in urine [18], most CSF proteins are found in the EVs they contain, as we have shown by a proteomic analysis of EVs versus total CSF and its proteins captured by the combinatorial peptide ligand library (CPLL), gathered through EVD from children with medulloblastoma [16]. Importantly, CSF proteomic studies have highlighted the vast dynamic protein range of CSF, against which the quantification of the low-abundance proteins, which are thought to be relevant to disease, is hampered, especially with label-free methods [16].

There are limitations in the use of CSF as a biomarker source e.g., age-related composition, low amounts, invasive sampling. Thus, achieving the necessary statistical power and obtaining robust control cohorts has been challenging [3]. Most studies have utilized CSF gathered by lumbar puncture. The lumbar site of access can cause blood contamination of the sample, and sample collection and storage lack reproducibility [6]. CSF can also be gathered through intra-operative sampling of the ventricles, which allows to overcome the scarcity of the sample and the contamination, in particular, using CSF from the extra-ventricular catheter (EVC). External drainage is used to divert CSF to avoid an increase in intracranial pressure in patients with hydrocephalus from several causes. CSF from EVD, routinely discarded as waste, is a viable source for analysis and biomarker discovery, and leverages procedures that are part of the routine clinical management of patients with hydrocephalus associated with a wide range of CNS conditions (e.g., malformations, brain tumors, and neurodevelopmental disorders). 

In collaboration with the neurosurgery department, we gathered and analyzed a rich set of CSF, obtained from EVD, of well-characterized patients with a variety of conditions. Our previous proteomic analysis of CSF from children with CNS tumors requiring EVD identified and validated potential biomarkers able to discriminate a tumoral from a non-tumoral condition and between control and medulloblastoma samples, suggesting that this analysis is a reliable method for biomarker discovery [14]. This study demonstrates that CSF proteomics and, in particular, the protein content of EVs can serve as potential sensitive and specific biomarkers in pediatric CNS tumors. Tumor biomarkers enable us to characterize the types of childhood brain tumors, providing insight into their pathogenetic mechanisms and assisting in monitoring treatment response and residual disease or recurrence, paving the way for risk-adapted therapeutic stratification and prognosis [14,15,16]. This is especially important in the case of pediatric brain tumors that are not resectable, where sensitive biomarkers would enable stratifying patients for non-surgical interventions. Similarly, disease stratification, prognosis, and treatment monitoring are common unmet needs in rare genetic neurodevelopmental disorders and epilepsies. These conditions are often caused by mutations in single (or multiple) CNS genes, which are often also found to be relevant for neurooncological and neurodegenerative disorders. The purpose of this study was to identify potential protein biomarkers of oncologic and non-oncologic brain disease, leveraging on our previous proteomic deposited data from pediatric control CSF samples, centrally collected through EVD [2,14], and comparing the differential enrichment of potential biomarkers among soluble and EV (Ex and Mv) compartments. We expected to confirm that although CSF is an active window into the CNS, most of the viable biomarkers or therapeutic targets of CNS disease are lost unless EVs are studied. 

## 2. Materials and Methods

### 2.1. Patients

We fractionated the proteome profile of CSF from EVD of 6 individual controls plus 2 pooled samples by 3 different biophysical methodologies, obtaining its Mv and exosomes (Ex) fractions either unprocessed or in its current sample state (Tot). We also analyzed our previous MS data [16].

For the validation part of this study, we enrolled 10 patients with congenital hydrocephalus unrelated to a brain tumor (CTR), 10 patients with pilocytic astrocytoma (PA), and 10 patients with medulloblastoma (MB). The main demographic and clinical features are summarized in Table 1.

The study was performed according to the Declaration of Helsinki and approved by Italian and international ethical guidelines and by the local ethics committee (No. 18 of 31 October 2013, protocol No. 176, ethics committee of “G. d’Annunzio” University and ASL No.2 of Lanciano, Vasto, and Chieti, Italy).

### 2.2. Mass Spectrometry Profile

Proteome profile analysis was performed as previously described [16]. Briefly, 1 mL of the total fraction of EVD of CSF and Mv and Ex fractions obtained from 50 mL of EVD of CSF by sequential ultracentrifugation were denatured, reduced, and alkylated by adding iST-LYSE buffer according to the manufacturer’s instructions (PREOMICS, Planegg/Martinsried, Germany) and then processed with the Freedom EVO 100 (TECAN, Mannedorf, Switzerland) using the protein aggregation capture method [19]. The captured proteins were digested using a mixture of 0.7 µg trypsin and 0.3 µg LysC enzymes overnight at 37 °C with a 1:20 enzyme-to-protein ratio. The resulting peptides were estimated using the tryptophan assay [20] and desalted with the IST protocol [21]. Then, peptides were eluted with a 200 cm µPAC C18 column (Thermo Fisher Scientific, Waltham, MA, USA) and separated using a non-linear gradient of 5–45% solution of 80% *v*/*v* acetonitrile, 5% *v*/*v* dimethyl sulfoxide, and 0.1% *v*/*v* formic acid in 155 min at a flow rate of 350 nL/min. Samples were acquired with the Orbitrap Fusion Tribrid instrument, and MS data were processed with MaxQuant software version 1.6.4.0. In the processing, a minimum length of 6 amino acids was required for peptide identification. Furthermore, acetyl (protein N-term), oxidation (M), and deamidation (NQ) were selected as variable modifications, and the fixed modification was carbamidomethyl (C).

### 2.3. Western Blotting 

The antibody specificity for syntaxin-binding protein 1 (STXBP1) was evaluated by Western blot analysis. Aliquots of 20 µg of a pool of unprocessed EVD of CSF (Tot), microvesicle (Mv), and exosome (Ex) fractions obtained from CTR patients were dissolved in 2% *w*/*v* SDS, 10% glycerol, and 62.5 mM Tris-HCl at pH 6.8, separated by sodium dodecyl sulfate polyacrylamide gel electrophoresis (SDS-PAGE), and then transferred to a nitrocellulose membrane for 30 min at room temperature using Trans-Blot SD cell apparatus (Bio-Rad Laboratories, Hercules, CA, USA). The full-length membrane was blocked, rinsed, labeled, and detected with rabbit polyclonal anti-human STXBP1 antibody (purchased from Merck/Sigma-Aldrich, Darmstadt, Germania, code SAB2102330) diluted to 1:1000 in phosphate buffer solution (PBS) containing 0.05% *v*/*v* Tween-20 (PBS-T) and 3% (*w*/*v*) of bovine serum albumin (BSA). After rinsing in PBS-T, the membrane was incubated with goat anti-rabbit IgG HRP-conjugated antibody (purchased from Novus Biologicals/Bio-Techne, Minneapolis, MN, USA), diluted to 1:10,000 in PBS-T containing 1% *w*/*v* BSA. The chemiluminescence signal was acquired and quantified using ChemiDoc and Quantity One software version 4.6.8 (Bio-Rad Laboratories, Hercules, CA, USA). Gel electrophoresis of the same samples was used as a loading control and the image was digitized using a GS-800 Densitometer (Bio-Rad Laboratories, Hercules, CA, USA).

### 2.4. Enzyme-Linked Immunosorbent Assay (ELISA)

STXPB1 was quantified in the validation cohort of 10 CTR patients, 10 patients with PA, and 10 medulloblastoma (MB) patients using a homemade direct ELISA. Briefly, the Nunc MaxiSorp™ ELISA plate (Thermo Fisher Scientific, Waltham, MA, USA) was coated with 5 µg of solubilized Total, Mv, and Ex samples diluted in carbonate buffer and incubated overnight at 4 °C. Uncoated wells were used for background subtraction. Wells were washed three times with PBS and blocked with 3% BSA in PBS overnight at 4 °C. Wells were washed again three times in PBS-T followed by incubation with 100 µL of rabbit polyclonal anti-human STXBP1 (purchased from Merck/Sigma-Aldrich, Darmstadt, Germania, code SAB2102330) diluted to 1:1000 in PBS-T containing 3% *w*/*v* BSA overnight at 4 °C. Wells were washed again three times with PBS-T and incubated with goat anti-rabbit IgG HRP-conjugated antibody (purchased from Novus Biologicals/Bio-Techne, Minneapolis, MN, USA) diluted to 1:2000 in PBS-T containing 1% *w*/*v* BSA for 1 h at room temperature. Finally, wells were washed again three times with PBS-T and developed with 3,3′,5,5′-Tetramethylbenzidine liquid substrate (Bio-Rad Laboratories, Hercules, CA, USA). The reaction was stopped by adding sulfuric acid and the absorbance was measured at 450 nm using the iMark plate reader apparatus (Bio-Rad Laboratories, Hercules, CA, USA). The reference standards were run in triplicate. A box plot was used to visualize the protein intensity. The lower detection limit was determined as the lowest protein intensity that could be differentiated from blank.

### 2.5. Statistical Analysis of Mass Spectrometry Data

After log2 conversion, the identified proteins were filtered for 70% presence in at least one fraction. Then, missing values were imputed using a normal distribution, and the whole dataset was normalized using the quantile method. The normalized dataset was analyzed by unsupervised hierarchical clustering (multidimensional scaling with k-means) and Spearman’s correlation to identify outliers and dissimilarities between samples. An ANOVA test for unpaired samples was used to identify the proteins that distinguished the three fractions. Then, to identify the proteins that maximized the discrimination between Tot and Mv or Ex fractions, and between Mv and Ex fractions, we applied a *t*-test, machine learning methods such as a non-linear support vector machine (SVM), and partial least squares discriminant analysis (PLS-DA). For the ANOVA and *t*-test, proteins were considered statistically significantly expressed for power values of 80% and adjusted *p*-values ≤ 0.05 after correction for multiple interactions (Benjamini–Hochberg method). Volcano plots were used to visualize the *t*-test analyses, establishing the cutoff line using the function y  =  |c/(x −  x0)|. In addition, both the support vector machine (SVM) rank and variable importance in projection (VIP) score were used to identify a priority list of importance of statistically significant features in each discrimination. In the SVM ranking, the lowest value corresponds to the maximum discriminating power. By contrast, for the VIP score, the higher value corresponds to the maximum discriminating power. Furthermore, in SVM learning, a Kernel linear algorithm with a fourfold cross-validation approach was applied to estimate the prediction and the classification accuracy. The whole matrix was randomly divided into two parts: one for learning (65%) and the other for testing (35%). This partition was repeated until all samples had been used for both learning and testing, ensuring a comprehensive evaluation of the model’s performance across the entire dataset. The iterative nature of this process helps assess the generalization capabilities of the machine learning model and identify the prediction accuracy. Finally, the confusion matrix was used to identify the minimum number of variables (proteins) needed to distinguish the different clusters studied and to define the most promising ranked list of potential biomarkers of this discrimination.

Gene set enrichment analysis was performed to construct a functional protein network based on Gene Ontology (GO) annotation terms extracted from the Gene Ontology Consortium (http://www.geneontology.org/ (accessed on 6 March 2023)). After loading the protein expression data of the whole dataset, each GO annotation term was assigned a ranked list. These ranks consider the number of proteins associated with each gene annotation term across all identified proteins, their average change, and the *p*-value after correcting with the False Discovery Rate (FDR) for multiple interactions. These rank values are between −1 and 1, corresponding to the minimum and maximum enrichment in each fraction of the group. In the two-dimensional scatter plot, the points that belong to the line passing between the points with coordinates (1x, 1y) and (−1x, −1y) represent equally enriched GO annotation terms. The distance of each point from this line is proportional to the increase in the enrichment of the annotation terms in one of the two groups. Furthermore, the list of statistically significant proteins from each comparison was loaded into the Joint Pathway Analysis function of the MetaboAnalyR 5.0 package [22] to identify the protein-associated biochemical pathways that maximize discrimination between each fraction comparison. Each pathway with an impact score greater than the corresponding expected value and a *p*-value twice <0.05 was considered statistically significant. Kinase enrichment analysis (KEA) was also performed for statistically significant proteins to identify kinases potentially involved in regulating the phosphorylation signal in each fraction [23]. Each kinome tree was visualized using the Coral app [24]. 

### 2.6. Statistical Analysis of ELISA Results

For the ELISA, the Kruskal–Wallis non-parametric unpaired test was used to assess the difference in the intensity of the potential biomarkers between the three clinical groups in each sample fraction. Results were expressed as medians and interquartile range (IQr). The receiver operating characteristic (ROC) curve was generated to assess the diagnostic efficiency of the assay in the discrimination between CTR and PA, and CTR and MB samples. The AUC value was classified as 0.5, not discriminant; 0.5–0.6, fail; 0.6–0.7, poor; 0.7–0.8, fair; 0.8–0.9, good; and 0.9–1, excellent. Youden’s index and the likelihood ratio were used to identify the cutoff and the diagnostic performance of the assay in each fraction, respectively. Two sides of *p*-values ≤ 0.05 were considered significant. All statistical tests were performed using Origin Lab Pro and the latest version of software package R available at the time of the experiments (R Core Team (2020). R: a language and environment for statistical computing. R Foundation for Statistical Computing, Vienna, Austria. URL https://www.R-project.org/ (accessed on 15 March 2023)).

## 3. Results

### 3.1. Proteins Profile

**Figure 1:**
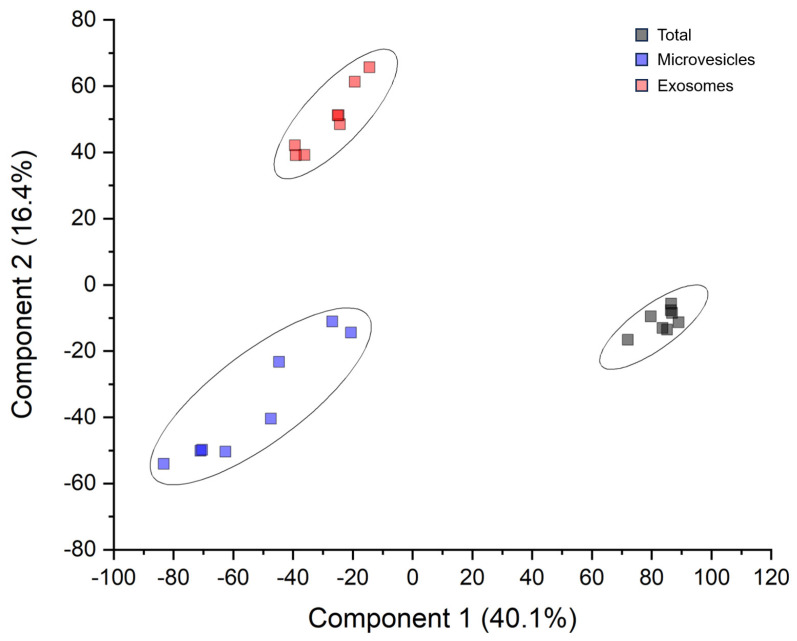
Multidimensional scaling with k-means of proteomic dataset of EVD of CSF fractions. Two-dimensional scatter plot of unsupervised cluster analysis of whole proteins dataset. Plot shows the absence of any outlier and the presence of three distinct clusters corresponding to total (black), microvesicles (blue), and exosomes (red) fractions.

A total of 1622 proteins, filtered for 70% of identity in at least 1 fraction, were identified, 1014 (62.5%), 1362 (84%), and 1065 (65.6%) of which were expressed in the unprocessed (Tot), microvesicles (Mv), and exosomes (Ex) fractions, respectively (Supplemental Table S1). Among these 1622 proteins, 629 (38.8%) were present in all fractions. Only 161 (9.9%), 199 (12.2%), and 32 (2%) proteins were exclusive of the Total, Mv, and Ex fractions, respectively (Supplemental Figure S1). Interestingly, out of 1622 identified proteins 1430 (88%), 1144 (70.5%), 233 (14.4%), 34 (2.1%), and 623 (38.4%) were annotated as associated with nervous system diseases, CNS neoplasm, synapse, synaptosome, and epilepsy disease, respectively. Furthermore, 23 (1.4%), 101 (6.2%), and 187 (11.5%) of total proteins were kinases, cluster differentiation molecules, and MEROPS, respectively. Despite a considerable overlapping of protein identity between the three fractions, multidimensional scaling analysis evidenced clear discrimination of three clusters corresponding to each fraction (Figure 1). 

**Figure 2:**
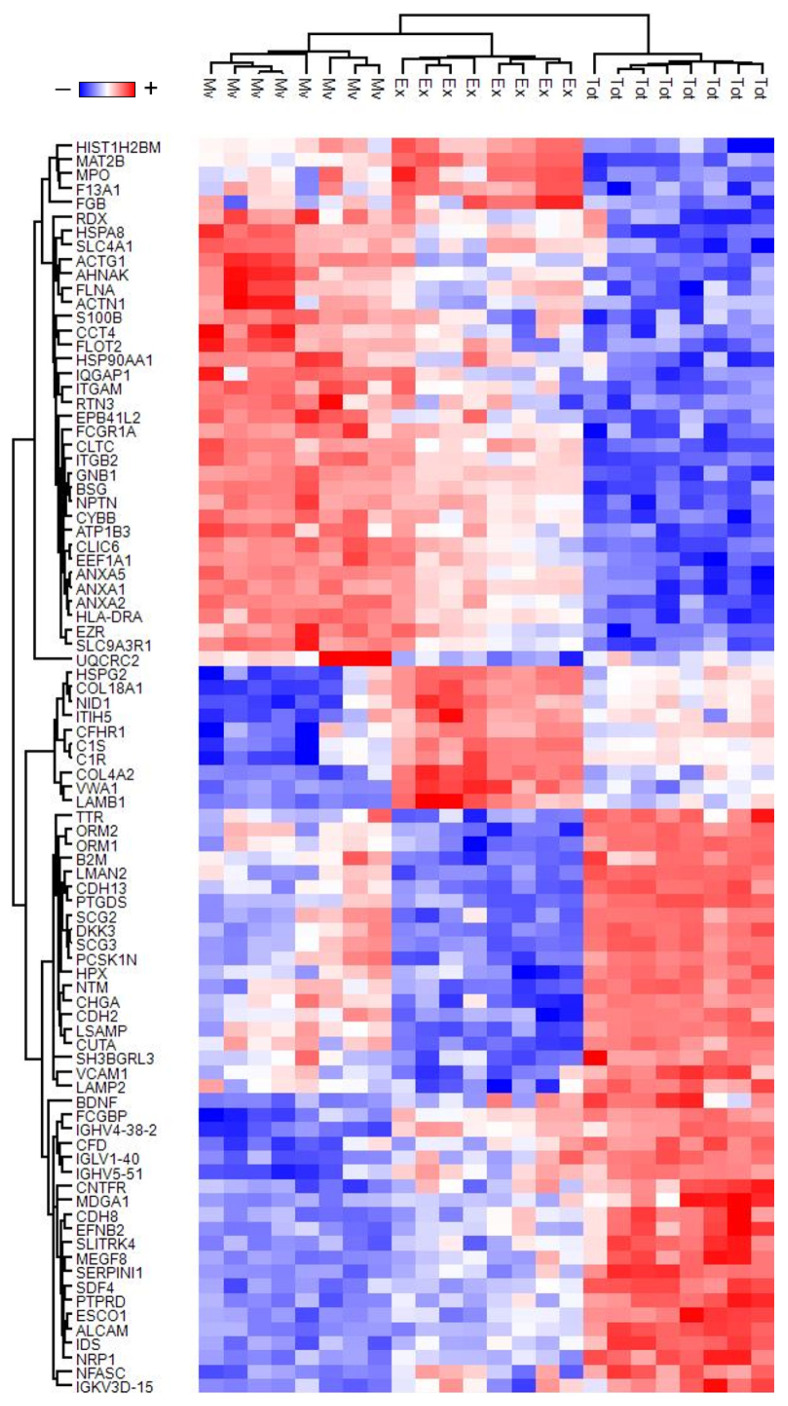
Heatmap of high discriminant proteins of three different fractions. Heatmap of 88 proteins highlighted by the combined use of ANOVA test and machine learning algorithm. In the heatmap, each row represents a protein, and each column corresponds to a sample. Normalized Z-scores of protein abundance are depicted by a pseudocolor scale with red indicating positive expression, white equal expression, and blue negative expression compared to each protein value, whereas the dendrogram displays the outcome of unsupervised hierarchical clustering analysis, placing similar protein profile values near each other. Visual inspection of the dendrogram and heatmap demonstrates the ability of these proteins to distinguish between total (Tot), microvesicles (Mv), and exosomes (Ex) fractions (see detail in Supplemental Table S1).

An ANOVA test for unpaired samples was used to identify the proteins that statistically distinguish between the three fractions. A total of 1015 proteins were identified (Supplemental Table S1). The expression profile of these proteins, after Z-score normalization, was visualized in the heatmap shown in Supplemental Figure S2. PLS-DA and SVM learning analyses were performed to reduce the number of selected proteins that maximize discrimination between the three fractions and to prioritize their importance. These analyses identified a ranked core panel of 88 proteins necessary for clear discrimination of the three fractions (Supplemental Table S2). Their priority was determined using the rank list and the variable importance in projection (VIP) score obtained using a SVM and PLS-DA, respectively. Both analyses identified the same protein priority order. Notably, the clear discrimination of the 3 fractions corresponds to a VIP score limit of 9.1. The k-means analysis associated with PLS-DA showed the presence of three distinct clusters, corresponding to the three fractions (Supplemental Figure S3). These results indicated that a SVM and PLS-DA are good models for the discrimination and prediction of samples (top position in both priority lists). The expression profile of the core panel of 88 proteins, after Z-score normalization, is visualized in the heatmap in Figure 2. Among these, the integration of statistical analyses and machine learning algorithms highlight the protein gene names AHNAK, ANXA2, ANXA5, BSG, GNB1, LAMB1, and MAT2B as the most promising potential biomarkers to distinguish the three different fractions. Indeed, these proteins show the highest -Log10 *p*-values after correction for multiple interactions in the ANOVA test and VIP score, and the lowest values in SVN rank. Furthermore, in receiver-operating characteristic (ROC) analysis, these proteins also show a statistically significant area under the curve (AUC) value equal to 1 in the discrimination of each comparison between sample fractions.

Then, a *t*-test was applied to identify the proteins that distinguish between Tot and Mv or Ex fractions. Out of 1622 proteins identified, 937, 578, and 295 were statistically significant in the comparison of Tot vs. Mv, Tot vs. Ex, and Ex vs. Mv fractions, respectively (Supplemental Table S1). Out of 937 statistically significant proteins in the comparison of Tot and Mv fractions, 453 and 484 proteins were upregulated in Mv and Tot fractions, respectively. Among these 937 statistically significant proteins, machine learning selected ICOSLG, GNAI2, ATP1A1, TUBB4B, SEZ6L2, ANXA6, MCFD2, and SLITRK1 as the most discriminative proteins between Tot and Mv fractions. Indeed, these proteins show the highest −Log10 *p*-values after correction for multiple interactions in the *t*-test and VIP score, and the lowest values in SVN rank. Furthermore, these proteins also show in ROC analysis a statistically significant AUC value equal to 1 in the discrimination of 2 sample fractions. Whereas, out of 578 statistically significant proteins in the comparison of Tot and Ex fractions, 235 and 343 proteins were upregulated in Ex and Tot fractions, respectively. Among these 578 statistically significant proteins, machine learning selected ANXA6, SERPINA6, GPM6A, ARF3, LMAN2, SCG3, and DKK3 as the most discriminative proteins between Tot and Ex fractions. Indeed, these proteins showed the highest −Log10 *p*-values after correction for multiple interactions in the *t*-test and VIP score, and the lowest values in SVN rank. Furthermore, these proteins also showed in ROC analysis a statistically significant AUC value equal to 1 in the discrimination of 2 sample fractions Finally, out of 295 statistically significant proteins in the comparison of Ex and Mv fractions, 146 and 149 proteins were upregulated in Mv and Ex fractions, respectively. Among these 295 statistically significant proteins, machine learning selected LAMA5, BANF1, COL4A2, VWA1, UNC5B, EMILIN1, EMILIN2, CYB5B, CUTA, ANXA5, HMGB1, GNB1, and LSAMP as the most discriminative proteins between Ex and Mv fractions. Indeed, these up- or down-expressed proteins show the highest −Log10 *p*-values after correction for multiple interactions in the *t*-test and VIP score, and the lowest values in SVN rank. Furthermore, these proteins also show in ROC analysis a statistically significant AUC value equal to 1 in the discrimination of 2 sample fractions. Volcano plots were used to visualize all these results (Supplement Figures S4–S6). Furthermore, out of 34 proteins annotated as being associated with synaptosomes, 13 (SNAP23, GNAI2, GNAI3, GLUL, SLC1A2, SLC1A3, STXBP1, GNAS, GNB1, GNG5, GNG12, ITPR2, and MPP5) were statistically significantly upregulated in both EV fractions if compared to the Tot fraction. Notably, STXBP1 is a known marker of brain disease whose negative enrichment has been implicated in both brain tumors and epilepsy [17,25,26]. According to the integrated analysis, two G-proteins, GNB1 and GNAI2, were discriminative proteins between the three fractions in the healthy EVD of CSF. 

In summary, a total of 1051 statistically significant proteins were identified using the ANOVA test, of which 937 (89%), 578 (55%), and 295 (28%) were also statistically significant when comparing Tot vs. Mv, Tot vs. Ex, and Ex vs. Mv fractions, respectively. Among these 1051 statistically significant proteins, 88 (8.4%) were statistically significant in all comparisons. Only 266 (25.3%), 5 (0.5%), and 109 (10.4%) proteins were exclusively significant in the comparisons between Tot vs. Mv, Tot vs. Ex, and Ex vs. Mv fractions, respectively (Supplementary Figure S7).

### 3.2. Gene Ontology Enrichment Analysis

**Figure 3:**
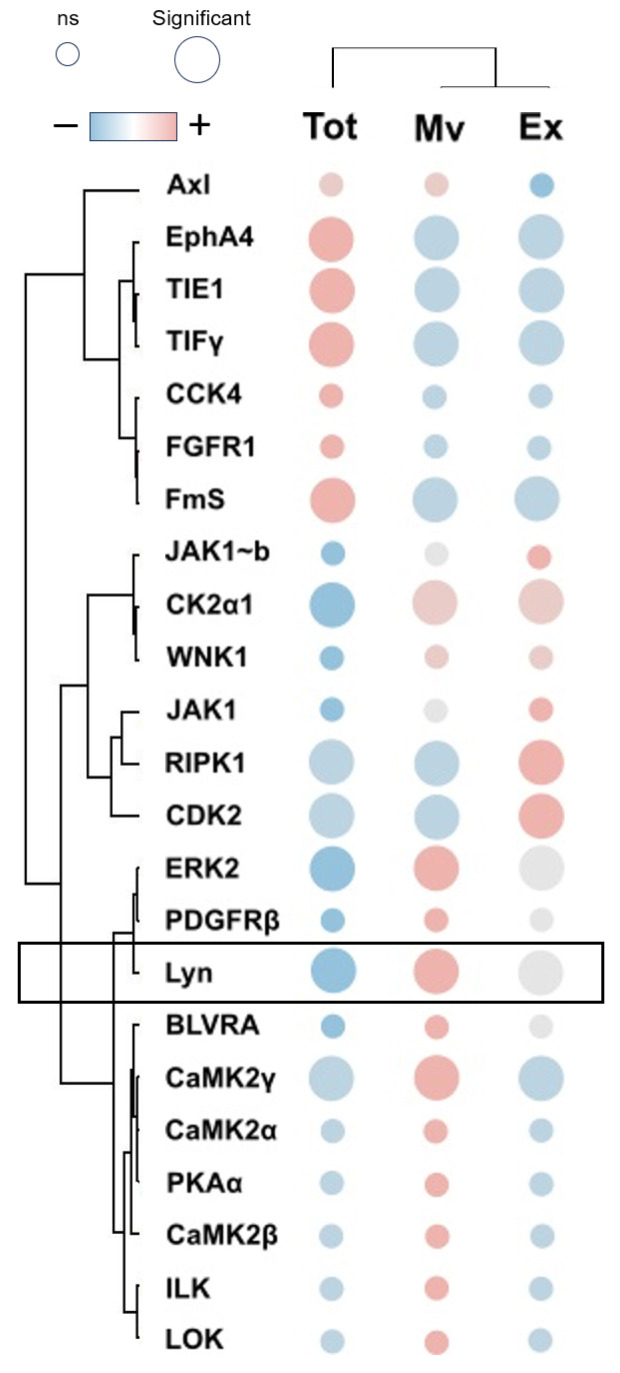
Heatmap of all kinases identified. Heatmap of 23 kinases identified by MS approach. In the heatmap, each row represents a kinase, and each column corresponds to a fraction. Normalized Z-scores of protein abundance are depicted by a pseudocolor scale with red indicating positive expression, white equal expression, and blue negative expression compared to each protein value, whereas the dendrogram displays the outcome of unsupervised hierarchical clustering analysis, placing similar protein profile values near each other. Visual inspection of the dendrogram and heatmap demonstrates the ability of Lyn kinase to distinguish between total (Tot), microvesicles (Mv), and exosomes (Ex) fractions (see details in Supplemental Table S1).

The considerable diversity in the expression profile of all the proteins identified in the three fractions may imply their different biological roles. To assess this, we performed a two-dimensional Gene Ontology (GO) enrichment analysis based on the annotation terms extracted from the Gene Ontology Consortium (http://www.geneontology.org/ (accessed on 6 March 2023)). This analysis identified 50 significantly enriched GO annotation terms in the comparison of Tot and Mv fractions. Among these, 23 and 27 were enriched above the 95% of CI in the Tot or Mv fractions, respectively (see details in Supplemental Table S3). Results are visualized by a scatter plot (Supplemental Figure S8), where each point corresponds to a GO annotation term. The points above or under the line with the equation x = y were, respectively, enriched in Mv or Tot fractions. Then, the same analysis applied to the comparison of Tot and Ex fractions identified 40 significantly enriched GO annotation terms. Among these, 24 and 16 were enriched above the 95% of CI in Tot or Ex fractions, respectively (see details in Supplemental Table S4 and Supplemental Figure S9). Finally, the same analysis applied to the comparison of Ex and Mv fractions identified 39 significantly enriched GO annotation terms. Among these, 30 and 9 were enriched above the 95% of CI in Ex or Mv fractions, respectively (see details in Supplemental Table S5 and Supplemental Figure S10). In summary, the results obtained from the above analysis showed, in both fractions of EV compared to the total fraction, an enrichment of proteins associated with tumor and non-tumor diseases of the CNS, such as epilepsy, of neuronal structures, such as the synaptosome, and of signal transduction. Furthermore, if the two extracellular fractions are compared with each other, the Mv are enriched in proteins associated with the synapses and especially with the GABAergic, serotonergic, cholinergic, and glutamatergic synapses, while the Ex are enriched in proteins associated with the regulation of the immune system, cell–cell and cell–matrix interaction and regulation of the cytoskeleton. Next, kinase enrichment analysis (KEA) [23] was conducted using all statistically significant proteins determined for each statistical analysis as substrates, plus the 23 kinases identified, to highlight the major kinases involved in the regulation of phosphorylation signaling in the 3 different fractions. Out of 23 kinases identified, 10 were statistically significant in at least 1 statistical comparison (Supplemental Table S1 and Supplemental Figures S11–S13). The expression profile of these kinases, after Z-score normalization, is visualized in the heat map shown in Figure 3. 

Then, gene names of all statistically significant proteins identified in this study with their expression profile and *p*-value were used for KEA analysis. This analysis predicted a total of 19 kinases statistically enriched in the 3 different fractions. Among these, only Lyn, FGFR1, and TIE1 were also identified (Supplemental Figure S14). Out of three kinases, TIE1 and Lyn results were statistically upregulated in the total fraction, and both EVs, respectively (with enrichment in Mv). On the contrary, the expression profile of FGFR1 was never statistically significantly changed. Furthermore, among these three kinases, only the variation in the expression profile of Lyn allows the discrimination of the three fractions.

**Figure 4:**
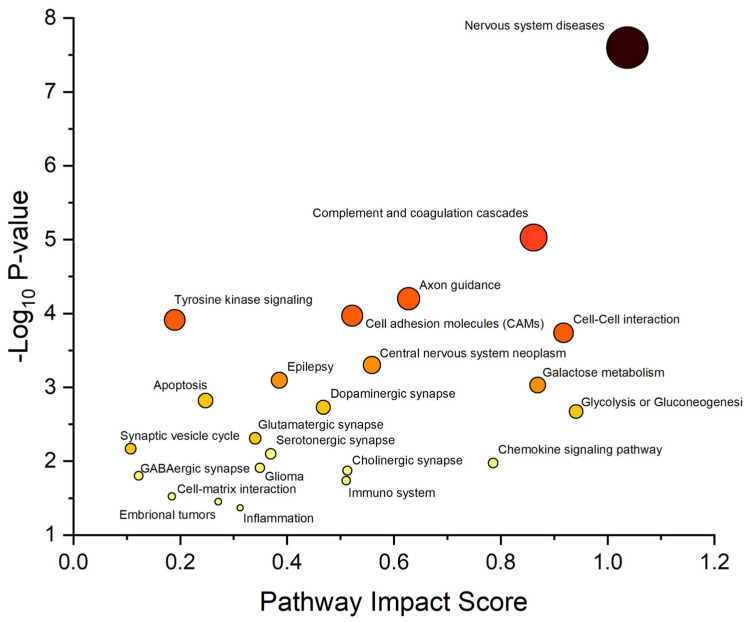
Gene Ontology (GO) enrichment analysis. Summary of EVD of CSF protein fractions GO analysis. In the plot, each circle represents a GO annotation term and the color is proportional to the corresponding *p*-value (the darker the color, the higher the *p*-value).

Finally, an integrated analysis of GO annotation terms was performed by combining the results obtained from KEA and two-dimensional enrichment analysis. This analysis revealed a total of 23 annotation terms associated with statistically significant changes in the protein expression profile between the three different fractions (Figure 4). Among these 23 pathways, 5, 5, 3, 2, and 2 can be grouped into CNS diseases, (CNS neoplasm, glioma, embryonal tumor, epilepsy, and axon guidance), synaptic (dopaminergic, glutamatergic, serotonergic, GABAergic synaptic, and synaptic vesicle cycle), molecular interaction (cell–cell interaction, cell–matrix interaction, and cell adhesion molecules), metabolism (glycolysis or gluconeogenesis, and galactose metabolism), and cell signaling (chemokine and tyrosine signaling). Considering all these results, many of the highlighted proteins appear closely related to non-tumoral and tumoral CNS diseases.

### 3.3. Western Blot Analysis

Primarily, the specificity of rabbit polyclonal anti-human STXBP1 antibody was tested with Western blot analysis. As shown in Supplemental Figure S15A, the antibody detected a single band corresponding to the molecular weight of the entire protein. Furthermore, the inability to detect the STXBP1 protein in the Tot fraction and its higher intensity in the Mv fraction compared to the Ex fraction appears confirmative of the results obtained in the MS-based approach. Blue silver staining [27] of the same samples was used as a loading control for the Western blot analysis (Supplemental Figure S15B).

### 3.4. ELISA Assays

**Figure 5:**
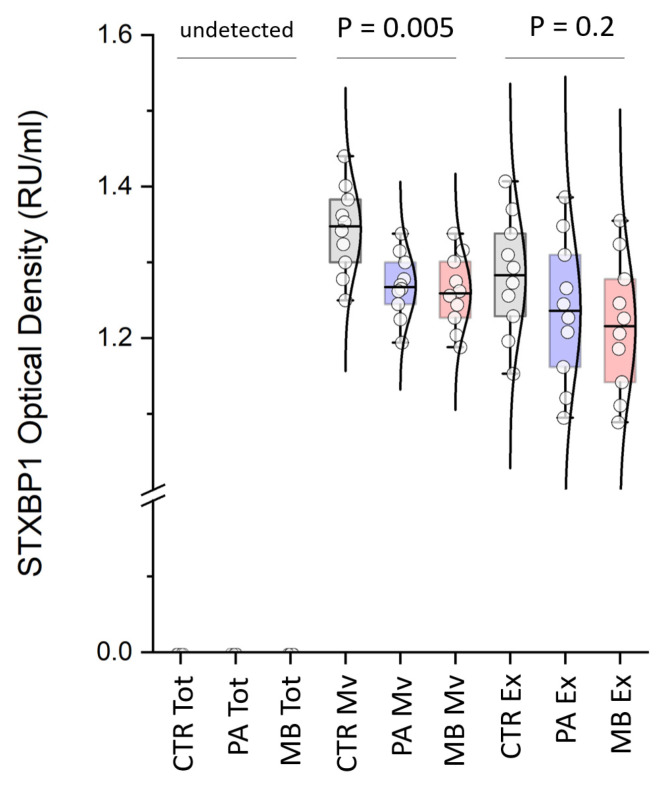
STXBP1 ELISA validation. Box plots of direct homemade ELISA assay for STXBP1 protein in unprocessed fraction (Tot) of EVD of CSF and its microvesicle (Mv) and exosome (Ex) fractions of congenital hydrocephalus (CTR), pilocytic astrocytoma (PA), and medulloblastoma (MB) patients. STXBP1 is undetected in Tot fraction, and more abundant in Mv (*p* = 0.005) and Ex (*p* = 0.2) of CTR compared to PA and MB patients.

Direct homemade ELISA assay was used to validate the potential role of a biomarker of STXBP1 protein expression in the EVs obtained from EVD of CSF of CTR, PA, and MB patients. Figure 5 shows that STXBP1 was differentially expressed in the EVs of CTR compared to PA and MB patients. In particular, in the Mv fraction, STXBP1 was statistically more abundant (*p* = 0.005) in CTR compared to PA and MB patients, with values of 1.35 (1.29–1.39), 1.27 (1.24–1.3), and 1.3 (1.26–1.34) Relative Units per milliliter (RU/mL), respectively. Furthermore, in the comparison of the Mv fraction between CTR and all brain tumor patients, the Area Under the Curve (AUC), Confidence Interval (CI), and *p*-value values were 0.86, 0.72–1, and 0.001, respectively. Finally, the assay’s cutoff value, sensitivity, specificity, and likelihood ratio were found to be <1.32, 90%, 70%, and 3, respectively. In Ex, STXBP1 was more abundant but not statistically significant (*p* = 0.2) in CTR compared to PA and MB patients, with values of 1.28 (1.22–1.41), 1.24 (1.15–1.32), and 1.22 (1.13–1.29) RU/mL, respectively. In the Ex fraction, when comparing the CTR and all brain tumor patients, the analysis yielded an AUC, CI, and *p*-value of 0.68, 0.48–0.88, and 0.11, respectively. Finally, the assay’s cutoff value, sensitivity, specificity, and likelihood ratio were found to be <1.23, 55%, 80%, and 2.75, respectively. The results of our homemade ELISA agree with data in the most recent literature reporting the downregulation of the STXBP1 protein (together with other proteins) as a predictor of malignancy in astrocytoma and identifying in medulloblastoma the low expression of the STXBP1 gene as a worse risk marker in survival compared to the high expression group [25,26].

## 4. Discussion

This study aimed at identifying the proteins enriched in CSF and its Ex and Mv fractions in search for biomarkers of brain disease. Sampling of CSF from control children needing an EVD for hydrocephalus allowed us to overcome both the ethical issues related to its collection and the sample volume limitations related to CSF collection through LP. Additionally, we decided to focus on STXBP1 as an example of a protein that is involved in both rare neurodevelopmental disorders [28], with epilepsy [17], and in pediatric CNS tumors [26].

Cerebrospinal fluid is the main component of the CNS extracellular space. Any brain pathology can alter the CSF proteome, which is therefore useful for discovering biomarkers of CNS diseases. However, individual CSF protein concentration spans an extraordinarily large (10 orders of magnitude) dynamic range. EVs do not display such high variability, being enriched fractions. Moreover, EVs may be more informative than CSF for those brain tumors that are not in close proximity to CSF. The bioinformatic study was conducted on EVD of CSF from normal subjects but, interestingly, results highlighted that Ex and Mv express proteins known in the literature as brain disease markers or therapeutic targets for CNS diseases, such as epilepsy, and CNS neoplastic diseases, such as gliomas, and embryonic tumors. The analysis of the 1622 proteins identified in the control CSF evidenced three discriminated clusters, corresponding to each fraction, and some proteins were able to better distinguish the three fractions. Among these there was basigin (or CD147), whose isoform 2 stimulates matrix metalloproteinases that play a key role in regulating tumor growth [29]. Prospective protein profiling of CSF from LP from 27 preterm infants showed that the proteome can predict neurodevelopmental outcomes [30]. In the proteomic study of CSF from children with the syndromic CLN3-Batten, a fatal neurodegenerative disease caused by mutations in the endolysosomal transmembrane CLN3 protein, a protein implicated in axonal development, were identified as candidate biomarkers [31].

Ex expresses the whole set of cell-to-cell communication proteins and tight junctions, consistent with their role of message carriers and the need to be guided to their target tissue. It can conceivably be assumed that Ex would be involved in metastasis: this was the case for melanoma [32] and retinoblastoma [33]. Mv, which appear to express the most significant biomarkers of brain disease, relay information about the CNS cell status, similar to what was described for Mv from preterm infants [34]. Ex is more enriched in proteins that function as cell-to-cell messages, while Mvs express proteins present on the plasma membrane, hence being representative of the interface between the cell and the external milieu. Consistently, the GO of the identified proteins in Mv were membrane proteins synapse, synaptosome, EVs, CNS neoplasm, nervous system disease, and epilepsy. The GO enriched in Ex were CNS neoplasm, synaptosome, epilepsy, and protein digestion. Furthermore, 33 out of the 34 synaptosome-related proteins (13 of which were increased) were expressed in the EVs. Among the 1622 identified proteins, 23 (1.4%) were tyrosine kinases. Lyn and ERK were the two kinases whose expression most statistically significantly varied. However, while Lyn is a tyrosine kinase belonging to the Src family involved in cancer development and neurodegenerative diseases [35], ERK is not predicted to be relevant in CNS disease pathways. Thus, the kinase predicted to be involved in driving the differentiation of the tumor-related pathways was also the one whose expression best differentiated the three fractions. This confirms that proteomic analysis of CSF EVs can give information on the ultimate orchestrators of the signaling pathways that lead to tumorigenesis. Notably, kinases are known therapeutic targets: drugs are available to modulate the action of the highlighted protein kinases. Lyn was undetected in the total CSF fraction, due to its concentration being too low, suggesting that the whole observation would be missed if EVs were not studied. For example, Supplemental Table S2 shows that beta-amyloid protein is enriched in the EVs, demonstrating that potential biomarkers of degenerative CNS disease are also present in EVs from normal CSF. The two biomarkers able to significantly distinguish the three different fractions of healthy EVD of CSF were the G-proteins Guanine nucleotide-binding protein subunit beta-1 (GNB1) and Guanine nucleotide-binding protein G(i) subunit alpha-2 (GNAI2), both modulators of transduction in transmembrane signaling systems and enriched in the MVs. Even though it was not among the highlighted portions, STXBP1 was validated as a key finding, as it is implicated in both brain tumors and epilepsy [17,25,26], being therefore not only a known biomarker of brain disease but also a potential target for therapy [17]. STXBP1 was among the 34 (2.1% out of the 1622 identified proteins) proteins annotated as associated with the synaptosome. STXBP1 is a presynaptic protein involved in neurotransmitter release [36,37] and the most frequent member of synaptosome-associated protein receptor superfamily (SNARE) complex-related genes involved in neurodevelopmental disorders and epilepsy [17,38]. STXBP1 maintains the integrity of the neuronal SNARE complexes that govern neurotransmission [39]. Interestingly, in our previous work on total CSF from EVD from various tumor types [3], STXBP1 was undetectable, as its prevalence in the total fraction was negligible. By contrast, STXBP1 was 100% detectable in Mv and 30–60% detectable in Ex, where it varied significantly. STXBP1 decreased significantly in MB samples, which represents a sign of malignancy [26]. Furthermore, the literature reports that STXBP1 is also involved in epilepsy, where its decreased function has been linked to the pathology [17]. Primary brain tumors are the most common childhood solid tumors and the leading cause of cancer-related mortality in children in all age groups [37,40,41]. The most diagnosed include pediatric low-grade gliomas (pLGG) accounting for up to 50% of pediatric CNS tumors, and embryonal tumors, among which is essentially MB and considered the most common malignant brain tumor in children [42]. Pediatric gliomas are categorized as low or high grade [43]. In particular, pilocytic astrocytoma (PA) is the most common type of pLGG [44]. PA is often caused by BRAF gene alterations, especially fusions [45], and has an excellent outcome after surgical resection with a low risk of high-grade progression, but there may be residual tumors [46]. MB is mostly localized in the cerebellum [47] with a peak incidence of around five years of age and is classified into four principal molecular groups (wingless activated (WNT), sonic hedgehog (SHH) activated, and non-SHH/WNT Group 3 and 4), according to the 2021 WHO Classification of Tumors of the CNS [48]. Advances in surgery, imaging, and adjuvant therapy have improved the survival rates of children with MB and pLGG, for which 5-year overall survival now exceeds 75% [36,42,49]. Again, STXBP1 was not expressed in the total CSF fraction sample from either a nontumoral or MB and PA, because its prevalence is too low. Interestingly, STXBP1 and PFKP were demonstrated to exhibit significant prognostic value in a study on 69 MB samples, and these proteins were inserted in a risk score model to predict the overall survival of MB patients [26]. Recently, it was shown that increased nuclear respiratory factor 1 (NRF1) transcription factor activity in astrocytoma is associated with poor survival [25]. NRF1 activity is associated with transcriptomic signatures of neurogenesis, cell stemness, and epithelial–mesenchymal transition, as well as downregulation of CASP3, STXBP1, and other potential biomarkers. These proteins predicted the extent of MB malignancy in high expressors of NRF1 activity. Significantly reduced expression of STXBP1 was found in autoptic brains of children with the pediatric neurodegenerative neuronal ceroid lipofuscinosis due to deficiency in palmitoyl protein thioesterase 1 (CLN1 disease) and is considered a candidate biomarker [50]. Variants in STXBP1 gene encoding for STXBP1 are common causes for neurodevelopmental disorders and epilepsy [51]. 

## 5. Conclusions

Considerable progress has been made in the last years in the understanding of the pathobiology of childhood oncologic [40] and non-oncologic brain diseases such as epilepsy [17]. However, deep knowledge of the molecular basis of these childhood conditions is still lacking. The present results suggest healthy CSF EVs express proteins already annotated as associated with nervous system diseases, CNS neoplasm, synapse, synaptosome, and epilepsy disease, where they can exclusively be identified. This is consistent with data showing that the most discriminating proteins of CSF from EVD of children with MB are enriched in the EV fraction [16]. To follow small variations in the putative marker expression, it needs to be significantly enriched, otherwise, no bioinformatic consideration can be performed. Utilizing EVs for “-omic” analyses of CSF from EVD would bypass the requirement of benchmarking the variability of the data on CSF proteome, which is required to correlate results to diseases. This is a pilot study that can, however, pave the way to subsequent studies that can establish the candidate biomarkers to specifically search for them in CSF in those cases where there is no indication of an EVD insertion. Should future studies show that the chosen biomarkers are also expressed in blood EVs, this would build the future liquid biopsy [52] for CNS disease for clinical applications of personalized medicine.

## Figures and Tables

**Table 1:** Clinical characteristics of all subjects present in the validation cohort of the study. All patients with brain tumors have a histological diagnosis. Age is reported as years (median and range).

Groups	Sex (F/M)	Age (Year)
**Controls**		
Congenital hydrocephalus (CTR)	5/5	2 (0–12)
**Low-grade Gliomas and Glioneural Tumors**		
Pilocytic Astrocytoma (PA)	5/5	8 (2–15)
**Embryonal tumors**		
Medulloblastoma (MB)	4/6	4 (0–14)

## Data Availability

The mass spectrometry data have been deposited to the ProteomeXchange Consortium via the PRIDE partner repository with the dataset identifiers. Project accession: PXD022512.

## Supplementary Materials

The following supporting information can be downloaded at https://www.mdpi.com/article/10.3390/biom13121730/s1, Figure S1. Venn diagram of all identified proteins. Figure S2. Heatmap of ANOVA statistically significant proteins. Figure S3. Partial Least Squire Discriminate Analysis (PLS-DA) of proteomic dataset. Figure S4. Volcano plot of comparison between total (Tot) and microvesicles (Mv) samples. Figure S5. Volcano plot of comparison between total (Tot) and exosomes (Ex) samples. Figure S6. Volcano plot of comparison between exosomes (Ex) and microvesicles (Mv) samples. Figure S7. Venn diagram of statistical analysis results. Figure S8. Two-dimensional Gene Ontology enrichment analysis between total and microvesicle fractions. FigureS9. Two-dimensional Gene Ontology enrichment analysis between total and exosome fractions. Figure S10. Two-dimensional Gene Ontology enrichment analysis between exosome and microvesicle fractions. Figure S11. Kinase tree diagram generated by the Coral app for the comparison between total and microvesicle (Mv) fractions. Figure S12. Kinase tree diagram generated by the Coral app for the comparison between total and exosome (Ex) fractions. Figure S13. Kinase tree diagram generated by the Coral app for the comparison between exosome (Ex) and microvesicle (Mv) fractions. Figure S14. Kinase tree diagram generated by Coral app for the predicted kinase involved in regulating phosphorylation transduction signaling among all statistically significant proteins (kinase enrichment analysis). Figure S15. Western blot analysis for STXBP1 protein in total fraction (Tot) of pooled EVD CSF and its microvesicle (Mv) and exosome (Ex) fractions of control (CTR) patients. Table S1. List of all identified proteins in extraventricular drainage of cerebrospinal fluid obtained from congenital hydrocephalus. Table S2. List of 88 proteins that maximize discrimination between the three different fractions. Table S3. Two-dimensional enrichment analysis between total and microvesicle fractions. Table S4. Two-dimensional enrichment analysis between total and exosome fractions. Table S5. Two-dimensional enrichment analysis between exosome and microvesicle fractions.

## Abbreviations

CNS	central nervous system
CSF	cerebrospinal fluid
EV	extracellular vesicle
EVD	extraventricular drainage
Ex	exosome
GO	Gene Ontology
KEA	kinase enrichment analysis
MB	medulloblastoma
Mv	microvesicle
PA	pilocytic astrocytoma
pLGG	pediatric low-grade glioma
STXBP	syntaxin-binding protein 1
SVM	support vector machine
PLS-DA	partial least squares discriminant analysis
VIP	variable importance in projection
