# Supplementary material for: Identification of Central Nervous System Oncologic Disease Biomarkers in EVs from Cerebrospinal Fluid (CSF) of Pediatric Patients: A Pilot Neuro-Proteomic Study
Source: Biomolecules. 2023 Nov 30;13(12):1730. doi: 10.3390/biom13121730 (PMC10741637; doi:10.3390/biom13121730)
Supplement: Supplementary file 1 [file biomolecules-13-01730-s001.zip › biomolecules-2709384-supplementary Figures.pdf]

SUPPLEMENTARY FIGURES

Identification of Central Nervous System Oncologic Disease Biomarkers in EVs from Cerebrospinal Fluid (CSF) of Pediatric Patients: A Pilot Neuro-Proteomic Study

Xhuliana Kajana [1], Sonia Spinelli [1], Andrea Garbarino [1], Ganna Balagura [2], Martina Bartolucci [3], Andrea Petretto [3], Marco Pavanello [1], Giovanni Candiano [1], Isabella Panfoli [4,*,†] and Maurizio Bruschi [1,5,†]

[1] Laboratory of Molecular Nephrology, IRCCS Istituto Giannina Gaslini, 16147 Genoa, Italy; soniaspinelli@gaslini.org (S.S.)
[2] Department of Neurosciences, Rehabilitation, Ophthalmology, Genetics, University of Genoa, 16132 Genoa, Italy
[3] Proteomics and Clinical Metabolomics Unit at the Core Facilities, IRCCS Istituto Giannina Gaslini, 16147 Genoa, Italy; martinabartolucci@gaslini.org (M.B.)
[4] Department of Pharmacy (DIFAR), School of Medical and Pharmaceutical Sciences, University of Genoa, 16132 Genoa, Italy
[5] Department of Experimental Medicine (DIMES), University of Genoa, 16132 Genoa, Italy
* Correspondence: isabella.panfoli@unige.it; Tel.: +39-0103537397
† These two authors contributed equally as the last author.

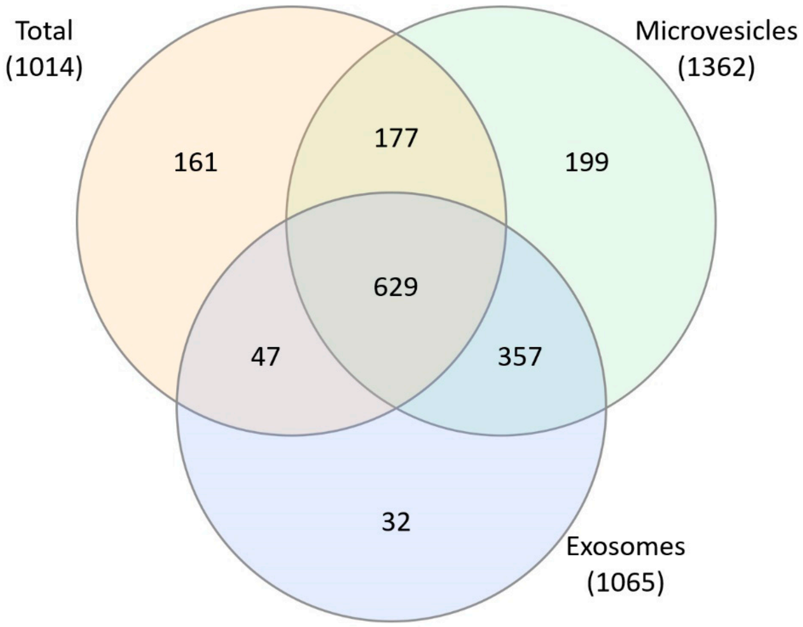

Figure S1. Venn diagram of all identified proteins. Venn diagram shows common and exclusive proteins. Numbers and circles represent the distinct proteins and the three different fractions, respectively.

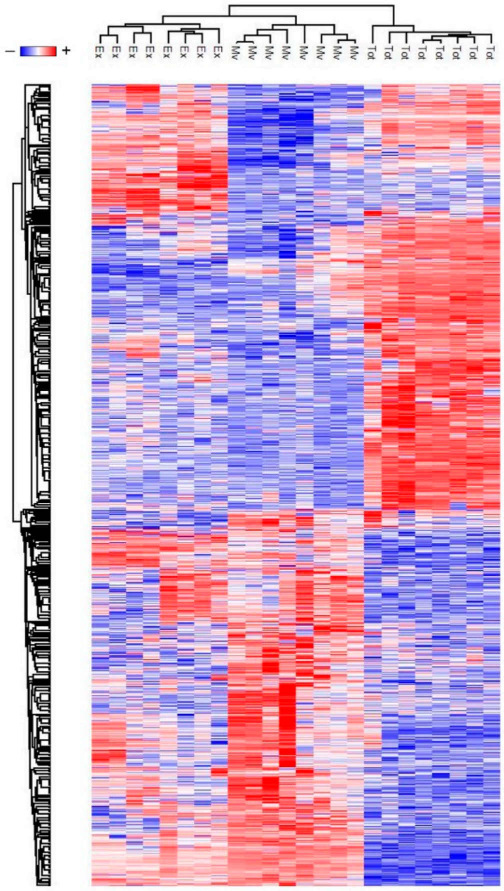

Figure S2. Heatmap of ANOVA statistically significant proteins. Heatmap of 1051 proteins highlighted using ANOVA test: in the heatmap, each row represents a protein, and each column corresponds to a sample. Normalized Z-scores of protein abundance are depicted by a pseudocolor scale with red indicating positive expression, white equal expression, and blue negative expression compared to each protein value. The dendrogram displays the outcome of unsupervised hierarchical clustering analysis, placing similar protein profile values near each other. Visual inspection of the dendrogram and heatmap demonstrates the ability of these proteins to distinguish between total (Tot), microvesicles (Mv), and exosomes (Ex) fractions (see detail in Supplemental Table 1).

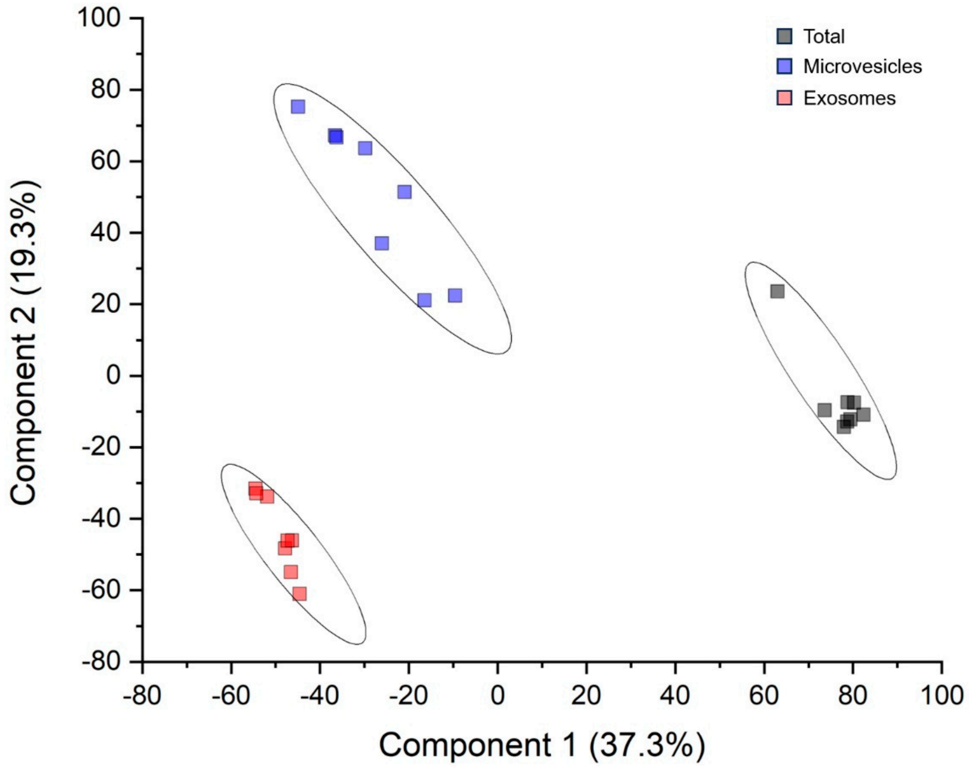

Figure S3. Partial Least Square Discriminant Analysis (PLS-DA) of proteomic dataset. Two-dimensional scatter plot of PLS-DA score of CSF EVD proteins profile label-free quantitation intensity from total (black), microvesicles (blue), and exosomes (red) fractions. Ellipses represent the 95% confidence intervals. The plot shows a clear separation of the three groups of samples.

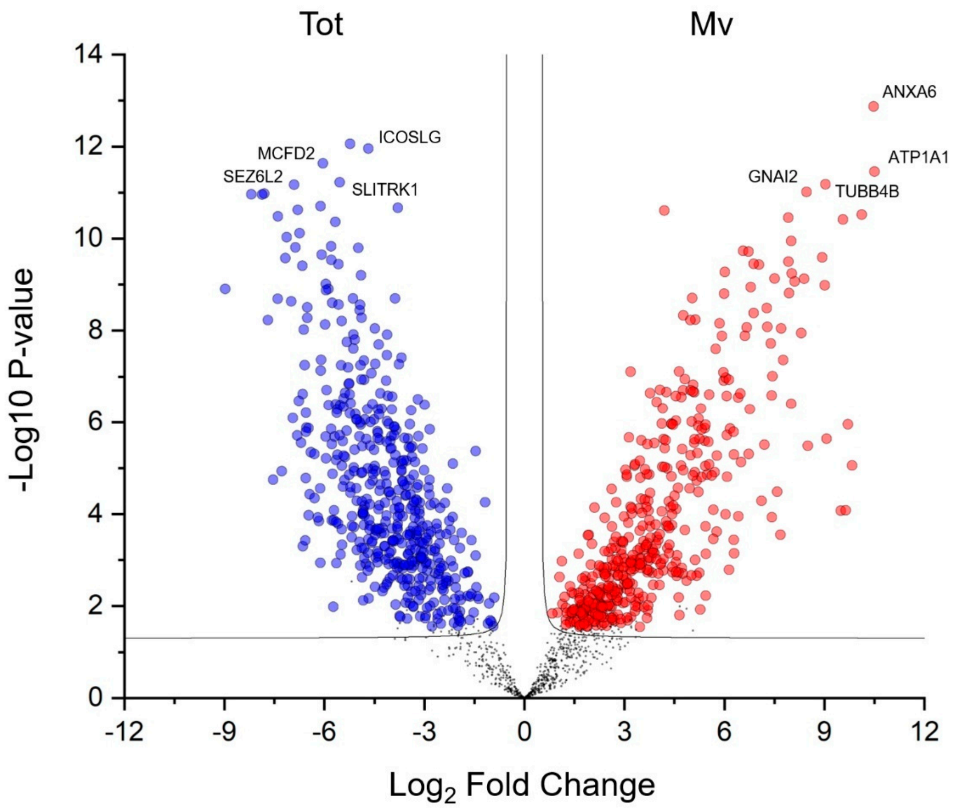

Figure S4. Volcano plot of the comparison between total (Tot) and microvesicle (Mv) samples. Graphical representation of T-test analysis applied to the whole proteomic dataset. The volcano plot x-axis and y-axis show the protein profile variation between Tot and Mv fractions and the corresponding P-value, respectively. The black curves show the statistical significance threshold. The plot shows the protein gene names that maximize the discrimination between Tot and Mv fraction obtained through the integration of T-test, partial least squire discriminant analysis and support vector machine learning algorithm.

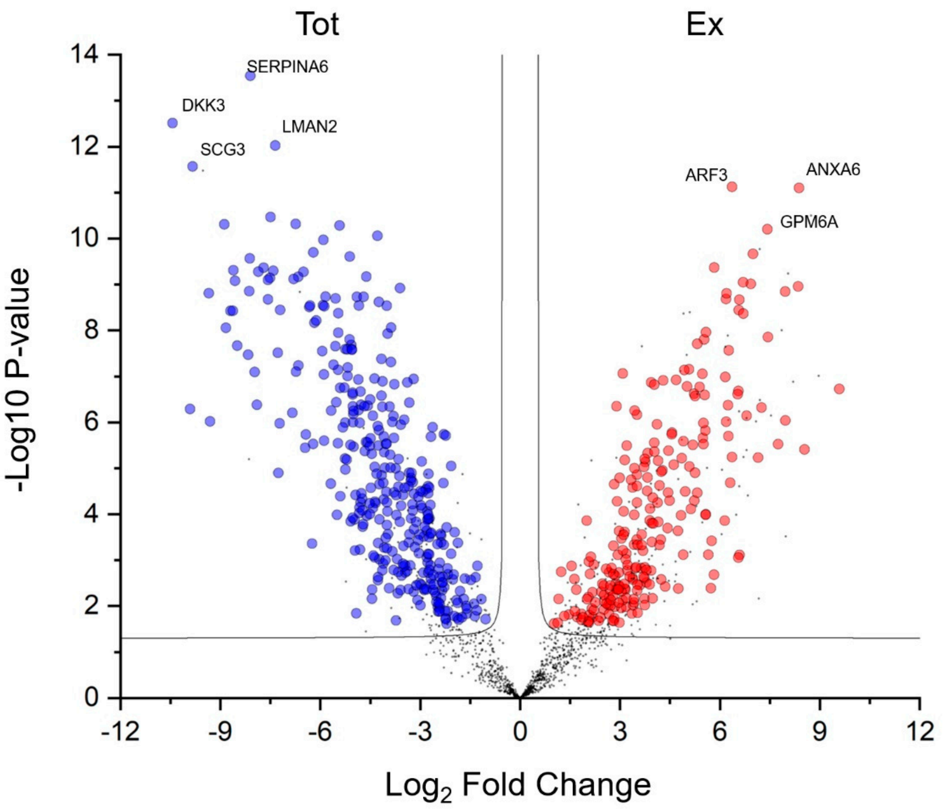

Figure S5. Volcano plot of the comparison between total (Tot) and exosomes (Ex) samples. Graphical representation of T-test analysis applied to the whole proteomic dataset. The volcano plot x-axis and y-axis show the protein profile variation between Tot and Mv fractions and the corresponding P-value, respectively. The black curves show the statistical significance threshold. The plot shows the protein gene names that maximize the discrimination between Tot and Ex fraction obtained through the integration of T-test, partial least squire discriminant analysis and support vector machine learning algorithm.

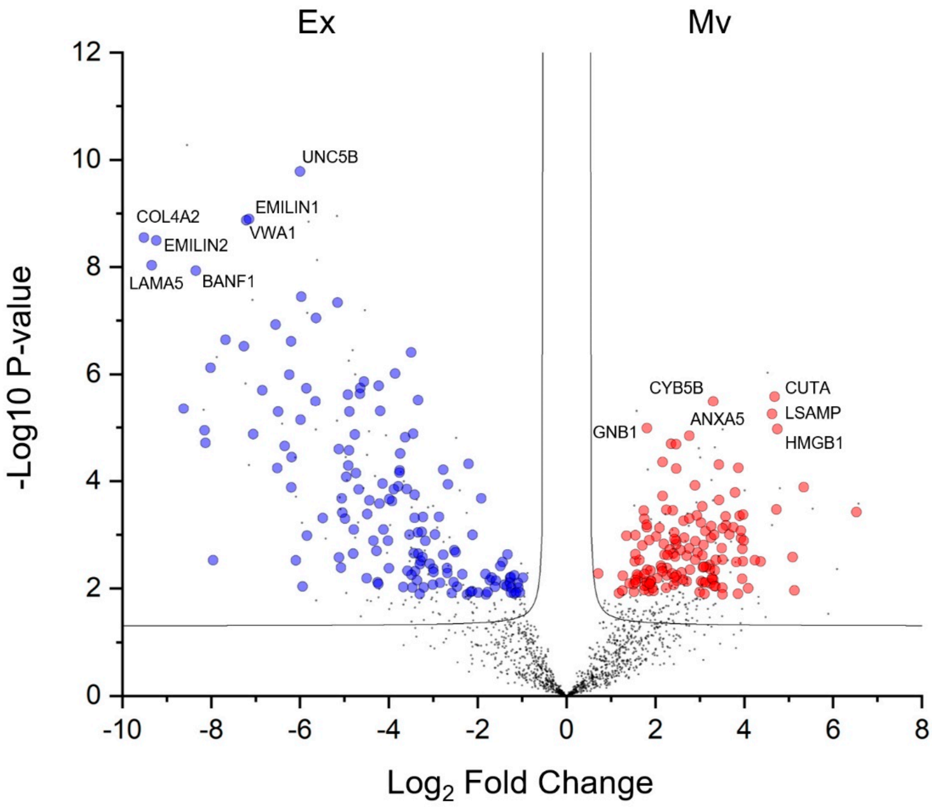

Figure S6. Volcano plot of comparison between exosomes (Ex) and microvesicles (Mv) samples. Graphical representation of T-test analysis applied at whole proteomic dataset. The volcano plot x-axis and y-axis show the protein profile variation between Tot and Mv fractions and the corresponding P-value, respectively. The black curves show the statistical significance threshold. The plot shows the protein gene names that maximize the discrimination between Mv and Ex fraction obtained through the integration of T-test, partial least squire discriminant analysis and support vector machine learning algorithm.

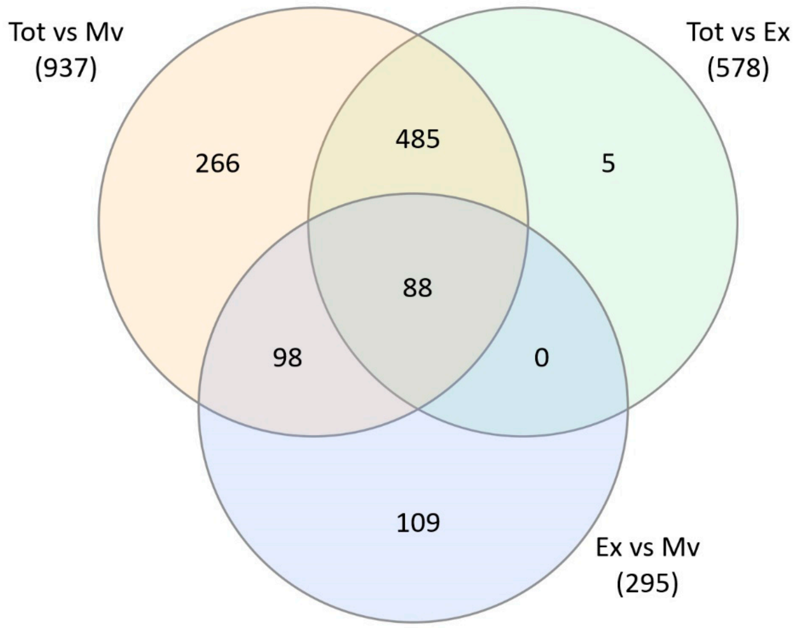

Figure S7. Venn diagram of statistical analysis results. Venn diagram shows common and exclusive proteins of the results of T-tests for all comparisons performed in this study. Numbers represent the distinct proteins in the respective overlapping and not-overlapping areas.

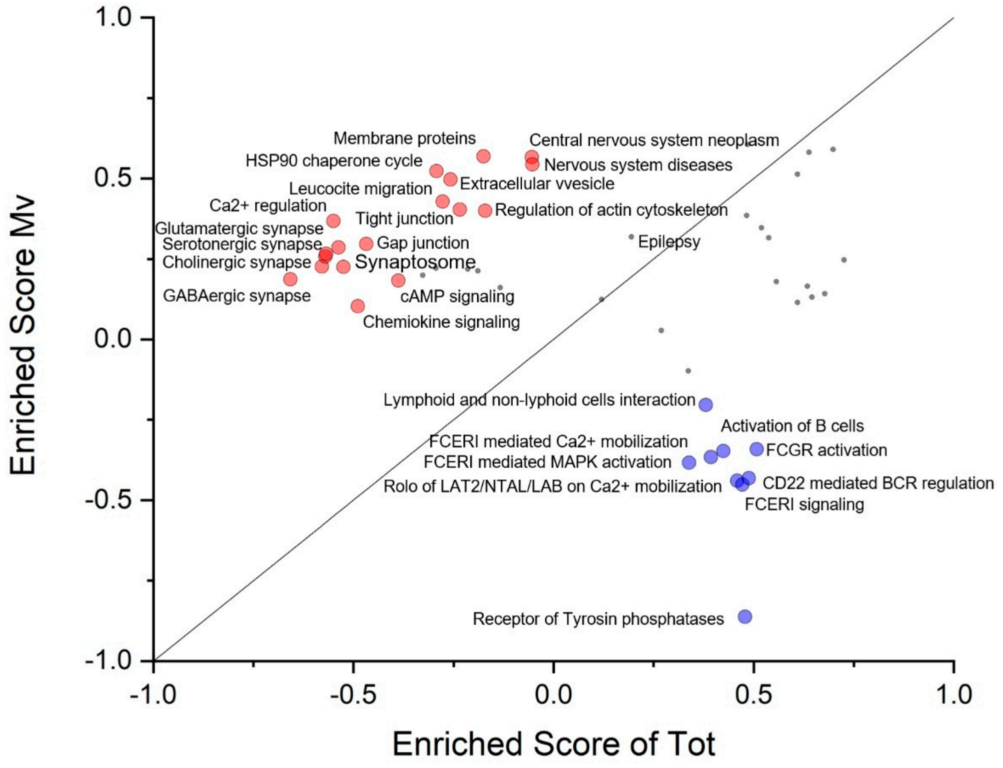

Figure S8. Two-dimensional Gene Ontology enrichment analysis between total and microvesicle fractions. The two-dimensional scatter plot shows the enriched signatures in the comparison between microvesicle (Mv) and total (Tot) fraction of CSF EVD of control samples. In the graph, the points located on the straight line passing through the coordinates (1x,1y) and (-1x,-1y) are the equally enriched signatures, while those above or under this line are positively enriched in Mv or Tot fractions, respectively. Red and blue circles are the signatures enriched above 95% of CI in Mv and Tot samples, respectively.

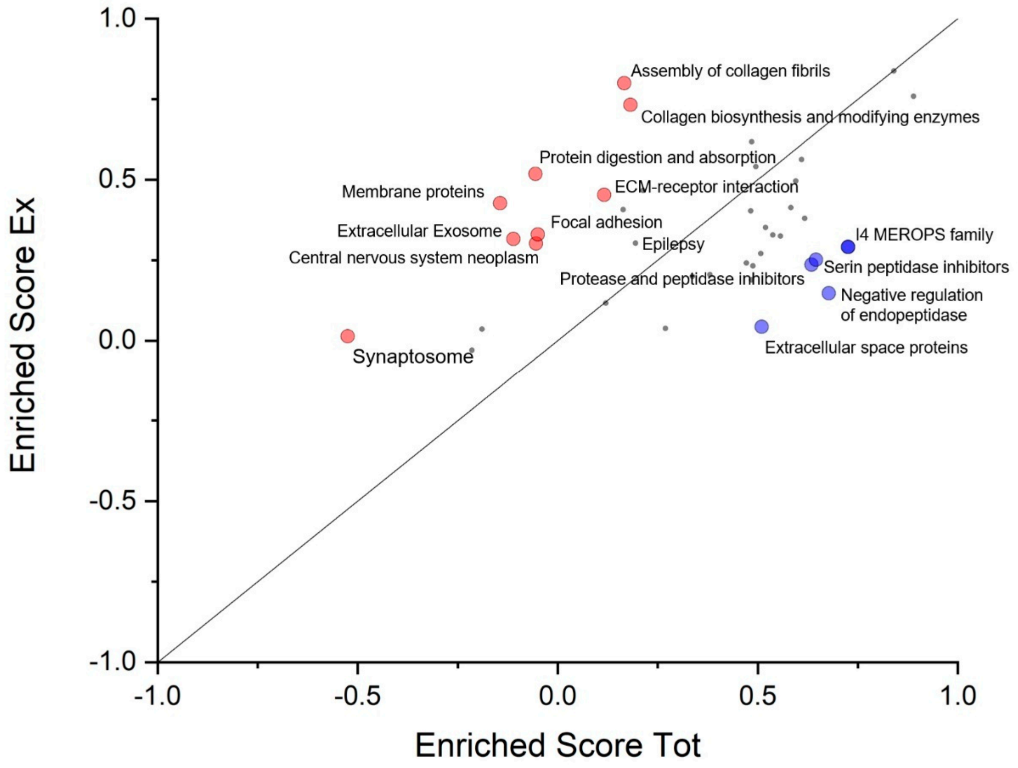

Figure S9. Two-dimensional Gene Ontology enrichment analysis between total and exosome fractions. The two-dimensional scatter plot shows the enriched signatures in the comparison between the exosome (Ex) and total (Tot) fraction of CSF EVD of control samples. In the graph, the points located on the straight line passing through the coordinates (1x,1y) and (-1x,-1y) are the equally enriched signatures, while those above or under this line are positively enriched in Ex or Tot fractions, respectively. Red and blue circles are the signatures enriched above 95% of CI in Ex and Tot samples, respectively.

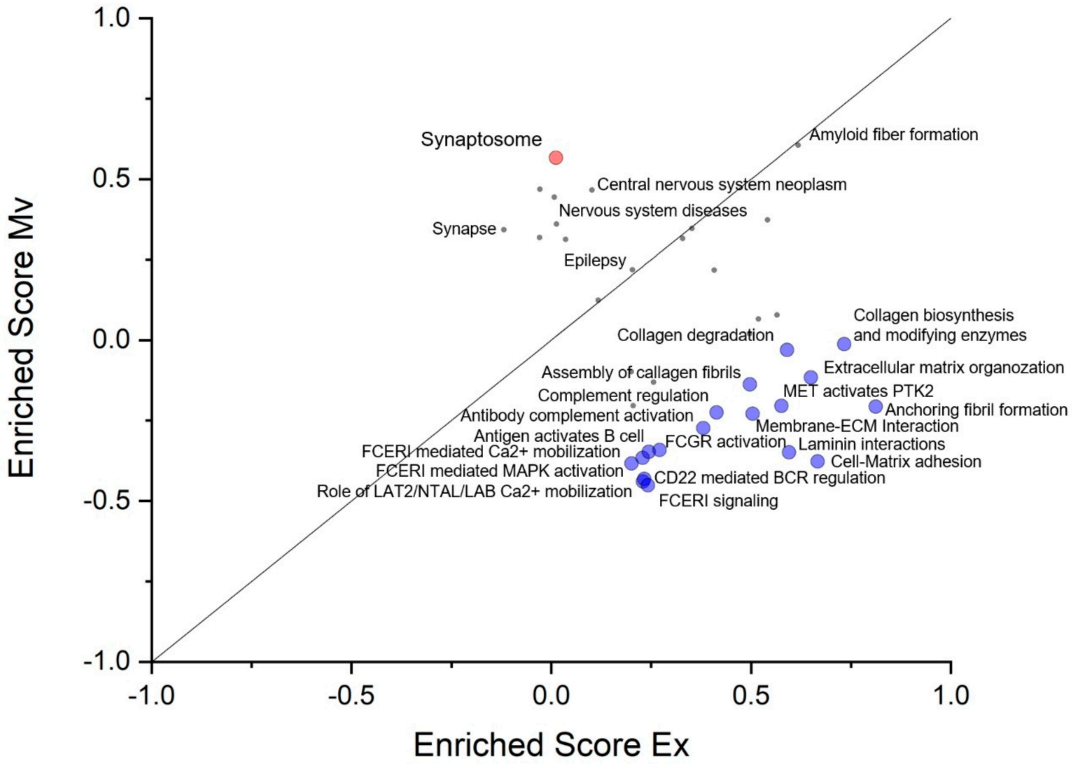

Figure S10. Two-dimensional Gene Ontology enrichment analysis between exosome and microvesicle fractions. The two-dimensional scatter plot shows the enriched signatures in the comparison between microvesicle (Mv) and exosome (Ex) fractions of CSF EVD of control samples. In the graph, the points located on the straight line passing through the coordinates (1x,1y) and (-1x,-1y) are the equally enriched signatures, while those above or under this line are positively enriched in Mv or Ex fractions, respectively. Red and blue circles are the signatures enriched above 95% of CI in Mv and Ex samples, respectively.

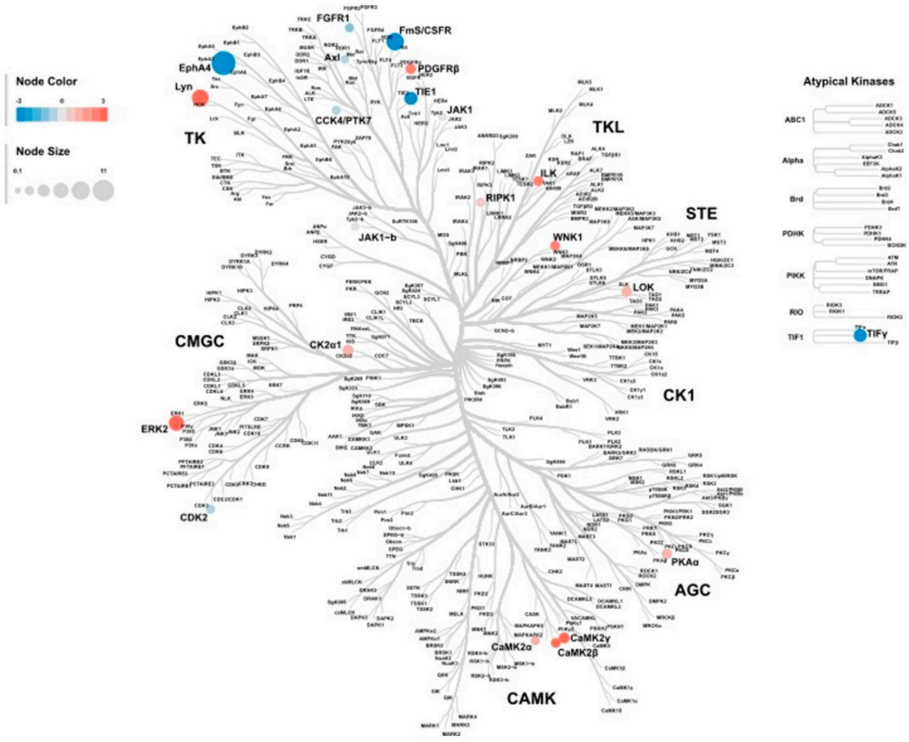

Figure S11. Kinase tree diagram generated by the Coral app for the comparison between total and microvesicle (Mv) fractions. Each circle represents an identified kinase. Log_2 kinase fold change is depicted by a pseudocolor scale with red indicating overexpression, white equal expression, and blue underexpression in Mv fraction. Circle size is proportional to corresponding $-Log_{10}$ P-value.

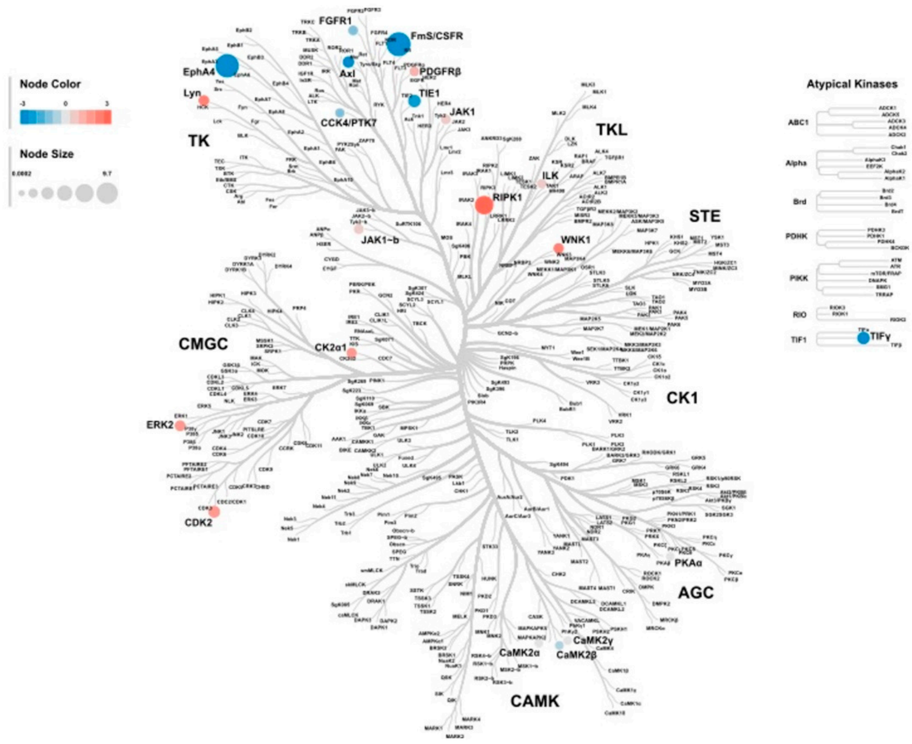

Figure S12. Kinase tree diagram generated by the Coral app for the comparison between total and exosome (Ex) fractions. Each circle represents an identified kinase. Log_2 kinase fold change is depicted by a pseudocolor scale with red indicating overexpression, white equal expression, and blue underexpression in Ex fraction. Circle size is proportional to the corresponding $-Log_{10}$ P-value.

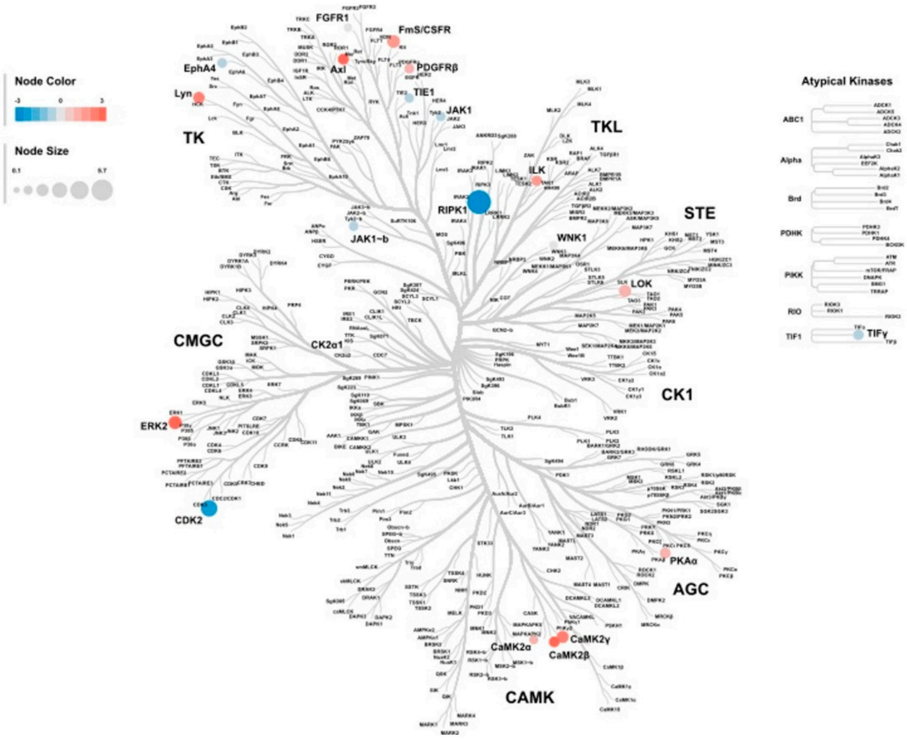

Figure S13. Kinase tree diagram generated by the Coral app for the comparison between exosome (Ex) and microvesicle (Mv) fractions. Each circle represents an identified kinase. Log_2 kinase fold change is depicted by a pseudocolor scale with red indicating overexpression, white equal expression, and blue underexpression in Mv fraction. Circle size is proportional to the corresponding $-Log_{10}$ P-value.

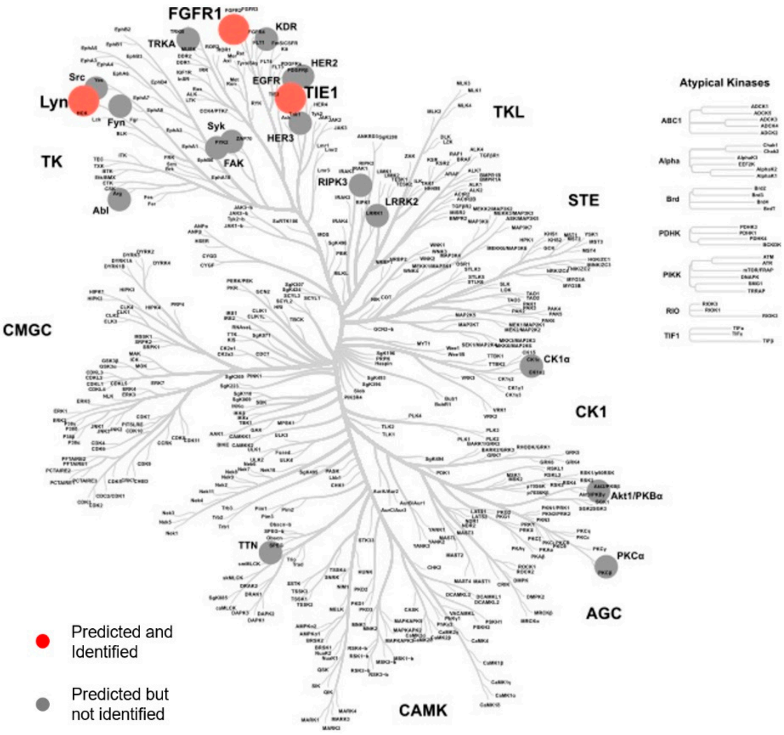

Figure S14. Kinase tree diagram generated by Coral app for the predicted kinase involved in regulating phosphorylation transduction signaling among all statistically significant proteins (kinase enrichment analysis). Each circle represents a predicted kinase with gray and red colors indicating its prediction and prediction and identification, respectively. Out of 19 predicted kinases, only three (FGFR1, Lyn, and TIE1) were predicted and identified in our mass spectrometry study. Among these Lyn is the only statistically significant discriminating kinase between the three fractions.

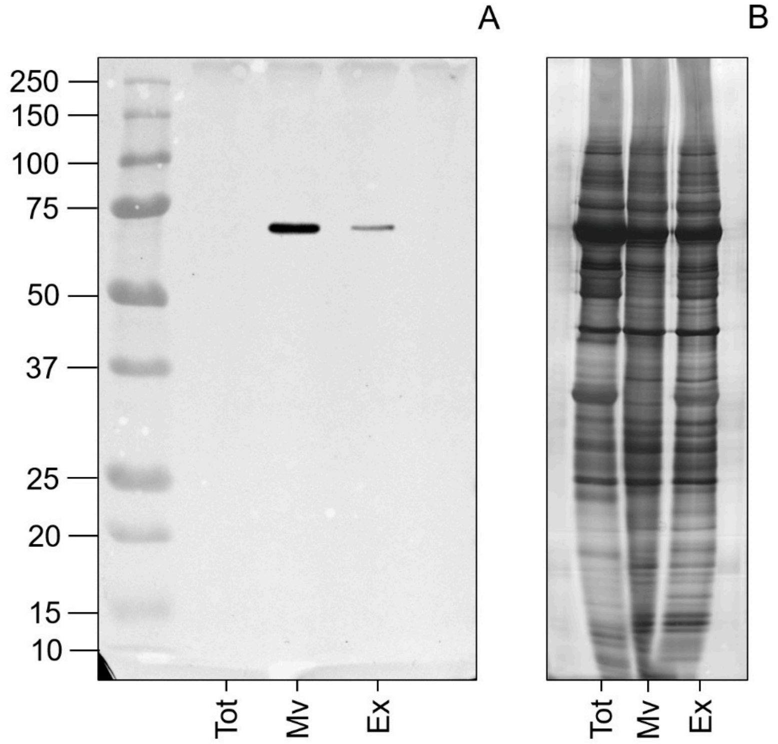

Figure S15. Western blot analysis. A) Representative western blot analysis of full-length gel (8-16T%) for STXBP1 protein in total fraction (Tot) of pooled CSF EVD and its microvesicle (Mv) and exosome (Ex) fractions of control (CTR) patients. STXBP1 is undetected in the Tot fraction and detected in a single band corresponding to the predicted molecular weight of the entire protein in Mv and Ex fractions. Furthermore, STXBP1 is more abundant in Mv fraction compared to Ex fraction; B) Blue silver staining of the same samples of the previous panel was used as loading control.
